# Supplementary figures and images for: Intracellular Salmonella Paratyphi A is motile and differs in the expression of flagella-chemotaxis, SPI-1 and carbon utilization pathways in comparison to intracellular S. Typhimurium
Source: PLoS Pathog. 2022 Apr 5;18(4):e1010425. doi: 10.1371/journal.ppat.1010425 (PMC9012535; doi:10.1371/journal.ppat.1010425)

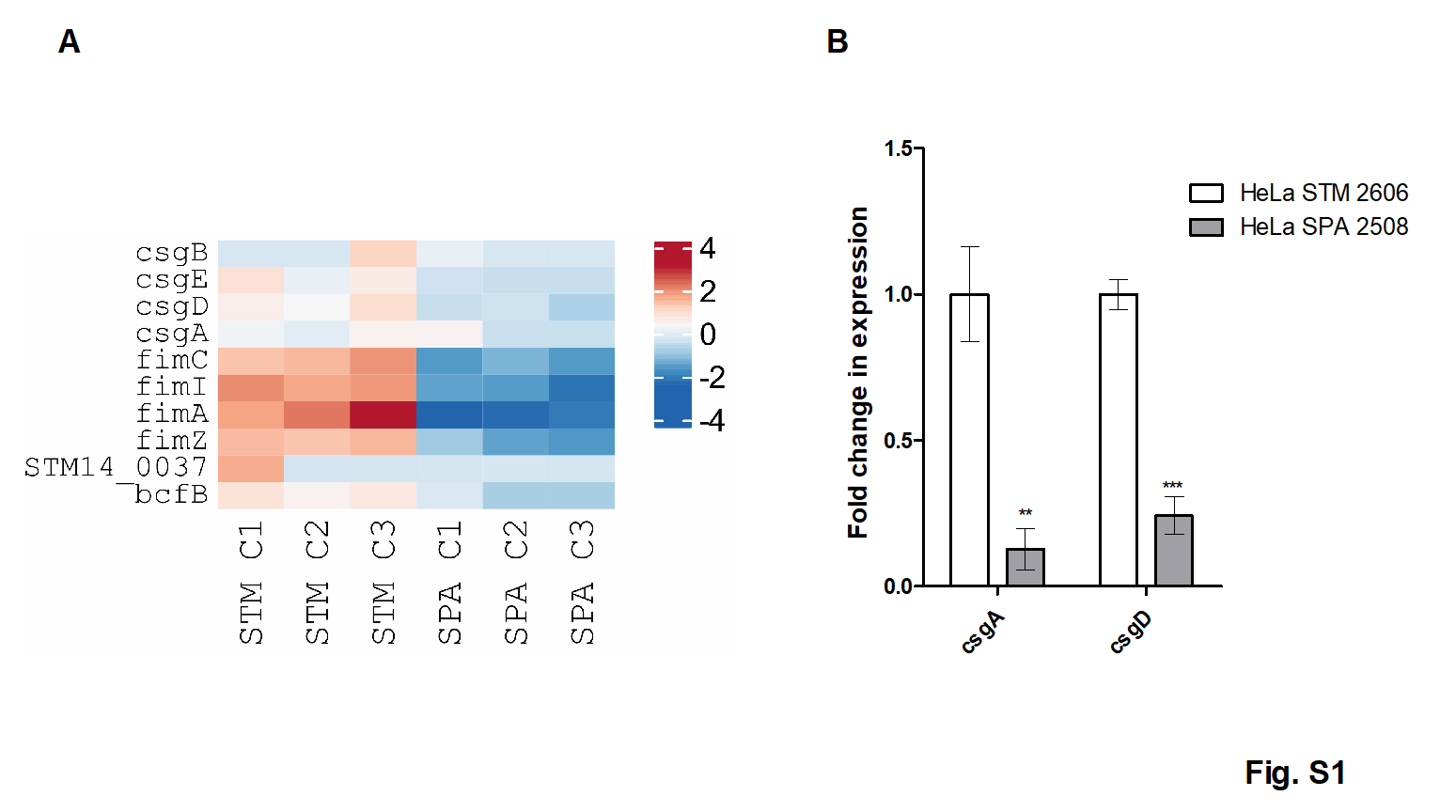

Supplement: S1 Fig — (A) A Heat map of RNA-Seq results showing the relative transcription of genes encoding curli (Csg), Fim, or Bcf fimbriae in three independent HeLa cells infections with STM and SPA. (B) The fold change in the expression of csgA and csgD in intracellular SPA relative to their expression in intracellular STM was analyzed by qRT-PCR. RNA was extracted from FACS-sorted intracellular salmonellae at 8 h p.i. The results show the means of 4–8 independent reactions and the error bars indicate the SEM. (TIF) [file ppat.1010425.s001.tif]

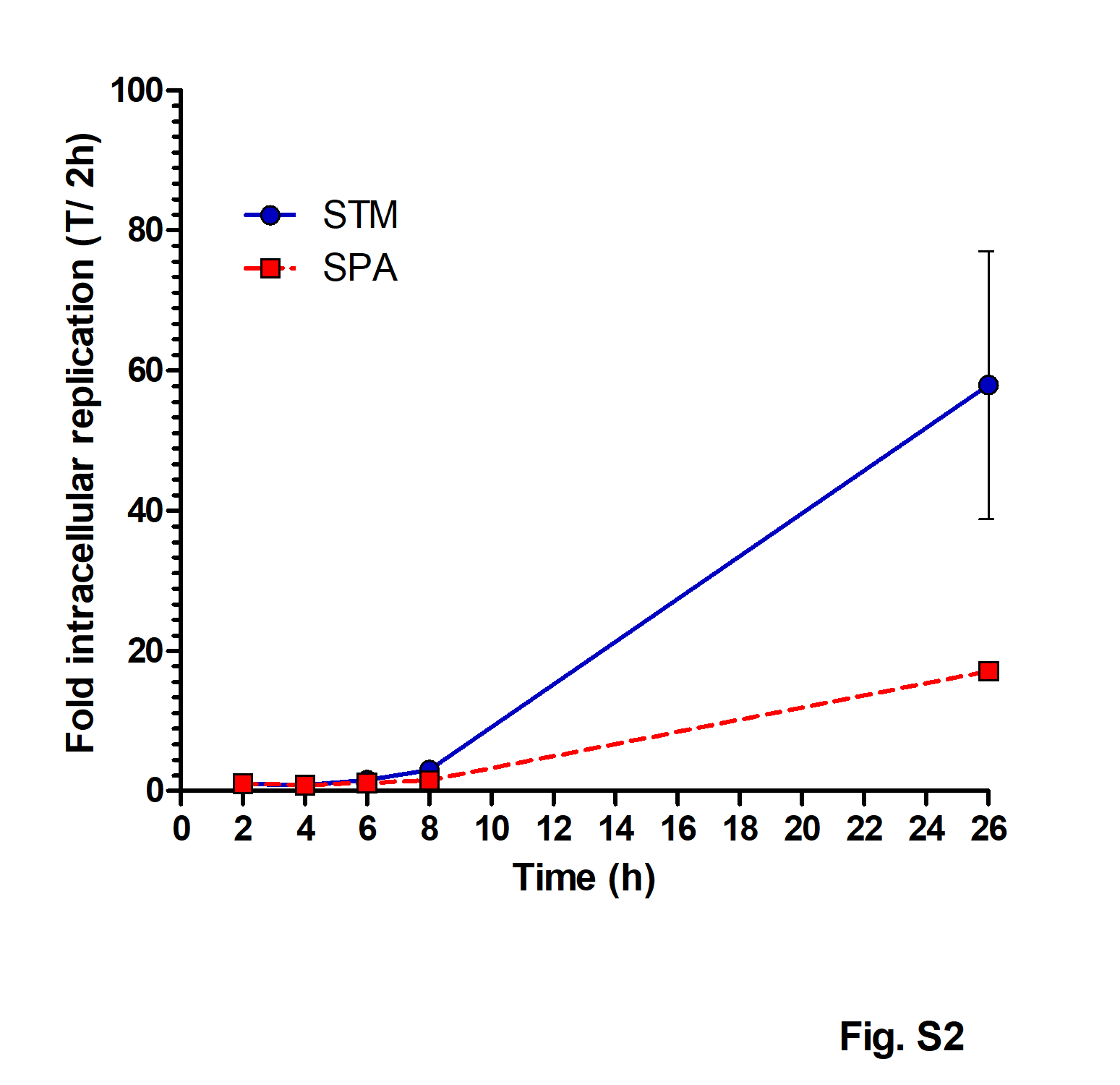

Supplement: S2 Fig — Intracellular growth of STM vs. SPA in HeLa cells at 2, 4, 6, 8 and 24 h p.i. Bacterial replication was determined by the gentamicin protection assay. Replication was calculated as the ratio between the intracellular bacteria (CFU) recovered at each time point and the number of CFU at 2 p.i. The means and the SEM of four independent infections are shown. (TIF) [file ppat.1010425.s002.tif]

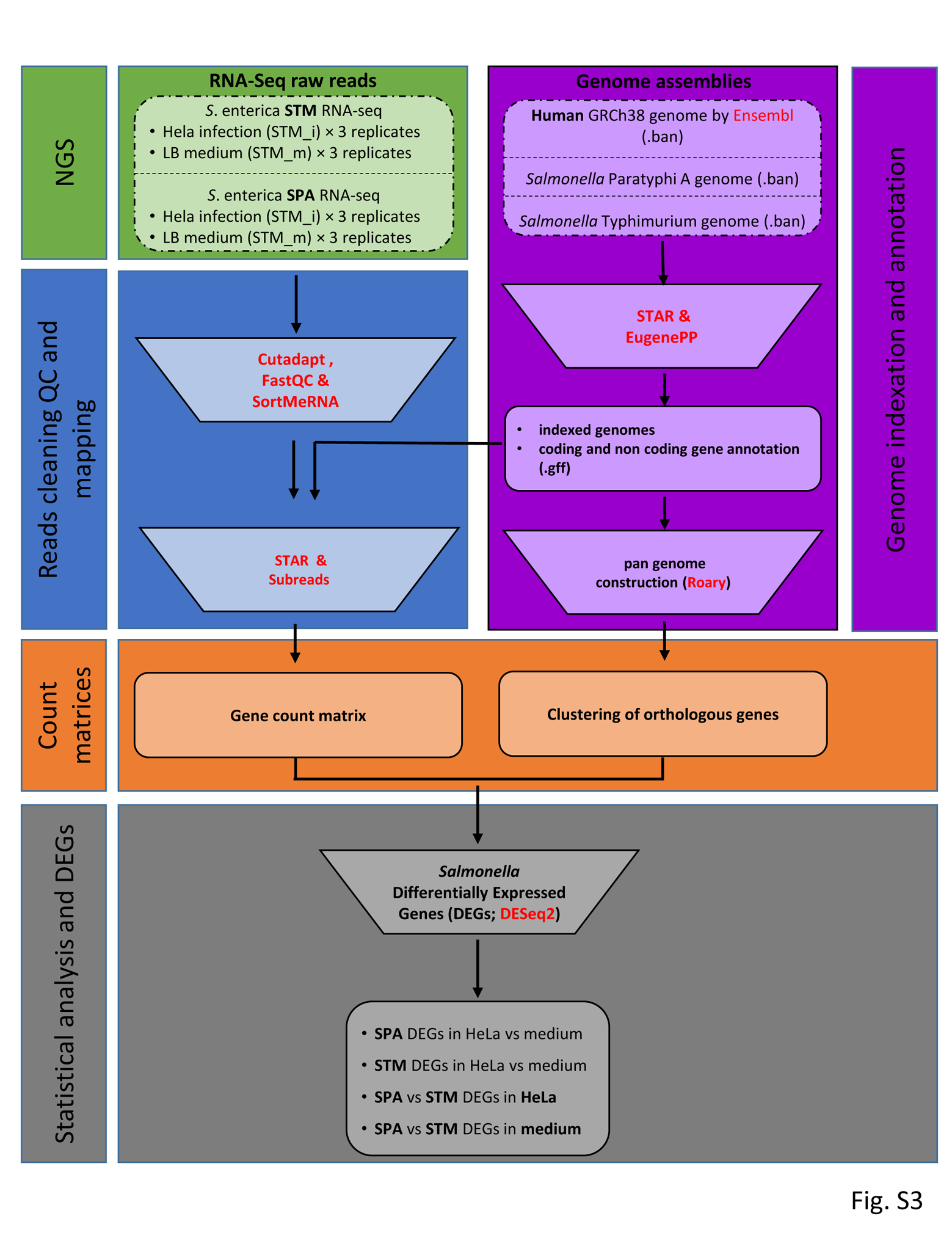

Supplement: S3 Fig — The different steps and main tools used for the bioinformatic analyses illustrated. RNA extracted from STM and SPA grown in culture and from infected cells was subjected to NGS on Illumina sequencing. RNA-Seq reads were cleaned and quality controlled using FastQC. Cutadapt was used to remove Illumina Truseq adaptors and to filter out all reads shorter than 36 nucleotides. SortMeRNA was used to filter out ribosomal RNA reads from raw data. Human and bacterial genomes were merged into “Hybrid genomes” and indexed with STAR. Bacterial genomes were reannotated using EugenePP. STAR was used to align reads to reference genomes and sort resulting bam files by coordinates. To compute fractional counting for all annotated coding and non-coding genes the Subread tool with parameters “-s 2 (reversely stranded), -p (count fragments instead of reads) -M, -O,—fraction” was used. Clustering of Salmonella homologous genes was computed using Roary. Normalization and differential expressed genes analysis were carried out according to the DESeq2 model and package using adjusted P-value and the Wald test to infer the probability value. To obtain DEGs between STM and SPA a count matrix composed of the shared and coding genes and ncRNAs between these two serovars was created. (TIF) [file ppat.1010425.s003.tif]
